# mRNA Abundance of Neurogenic Factors Correlates with Hearing Capacity in Auditory Brainstem Nuclei of the Rat

**DOI:** 10.3390/life13091858

**Published:** 2023-09-02

**Authors:** Jonas Engert, Julia Doll, Barbara Vona, Totta Ehret Kasemo, Bjoern Spahn, Rudolf Hagen, Kristen Rak, Johannes Voelker

**Affiliations:** 1 Department of Otorhinolaryngology, University Hospital Wuerzburg, Plastic, Aesthetic and Reconstructive Head and Neck Surgery, Josef-Schneider-Strasse 11, 97080 Wuerzburg, Germany; ehret_t@ukw.de (T.E.K.); spahn_b@ukw.de (B.S.); hagen_r@ukw.de (R.H.); rak_k@ukw.de (K.R.); voelker_j@ukw.de (J.V.); 2 Institute of Pathology, University of Wuerzburg, Josef-Schneider-Strasse 2, 97080 Wuerzburg, Germany; julia.doll@uni-wuerzburg.de; 3 Institute for Auditory Neuroscience and InnerEarLab, University Medical Center Göttingen, Robert-Koch-Strasse 40, 37075 Göttingen, Germany; barbara.vona@med.uni-goettingen.de; 4 Institute of Human Genetics, University Medical Center Göttingen, Heinrich-Düker-Weg 12, 37073 Göttingen, Germany

**Keywords:** real-time PCR, auditory pathway, neurogenesis, cochlear nucleus

## Abstract

Neural stem cells (NSCs) have previously been described up to the adult stage in the rat cochlear nucleus (CN). A decreasing neurogenic potential was observed with critical changes around hearing onset. A better understanding of molecular factors affecting NSCs and neurogenesis is of interest as they represent potential targets to treat the cause of neurologically based hearing disorders. The role of genes affecting NSC development and neurogenesis in CN over time on hearing capacity has remained unclear. This study investigated the mRNA abundance of genes influencing NSCs and neurogenesis in rats’ CN over time. The CN of rats on postnatal days 6, 12, and 24 were examined. Real-time quantitative polymerase chain reaction arrays were used to compare mRNA levels of 84 genes relevant to NSCs and neurogenesis. Age- and hearing-specific patterns of changes in mRNA abundance of neurogenically relevant genes were detected in the rat CN. Additionally, crucial neurogenic factors with significant and relevant influence on neurogenesis were identified. The results of this work should contribute to a better understanding of the molecular mechanisms underlying the neurogenesis of the auditory pathway.

## 1. Introduction

By 2050, approximately 700 million people worldwide will have hearing loss that requires therapy. In many patients, this is due to sensorineural hearing loss (SNHL), which is characterized by irreversible disease of structures of the inner ear or neural structures of the auditory pathway [1]. The development of new therapeutical techniques, such as the generation of human induced pluripotent stem cells or genome editing using CRISPR/Cas9, represent promising approaches for treating SNHL [2]. In recent years, effective genetic therapy options for the auditory system have already been developed in the rodent model [3]. CRISPR-Cas9 technology uses synthetic RNA specific to the target sequence [4]. Gene editing with CRISPR/Cas9 was found to prevent hearing loss in the Beethoven mouse model, which is a model for human non-syndromic autosomal-dominant deafness [5]. Another promising approach is RNA interference (RNAi), a naturally occurring posttranscriptional process of gene expression regulation [6]. A RNAi approach based on an artificial microRNA (miRNA) was shown to successfully avert progressive hearing loss in the Beethoven mouse model [7]. The effectiveness of these RNA-based approaches in translational models of hearing loss emphasizes the importance of molecular genetics for future hearing research [3].

NSCs are defined by their ability to undergo mitotic self-renewal, form progenitor cells, and differentiate into all cell types of the neuroectodermal cell lineage [8]. The subventricular zone and the hippocampus are prominent examples in which neurogenic capacity has been extensively studied [9]. NSCs have been detected along the auditory pathway of postnatal rats and mice in several nuclei up to the adult stage [10,11,12,13,14,15]. Their cardinal features have been characterized by in vitro studies using differentiation, immunohistology, and proliferation analysis. For the first time in the auditory system, NSCs were described in the spiral ganglion and stria vascularis [12,14]. Similarly, a NSC niche was detected in the auditory cortex, inferior colliculus, and medial geniculate body [10,11,15]. The cochlear nucleus (CN) exhibits NSC capacity into the adult stage. Here, a decreasing potential with increasing age was found in CN. The critical period from which proliferative capacity and expression of NSC markers began to decline was the time from postnatal days 10 to 14 (p10–p14). Interestingly, this corresponds to rat hearing onset [16]. From about p20, stem cell capacity was stable at a low level.

The CN is the first relay station of the auditory pathway. It harbors the cell bodies of the second auditory neuron. Furthermore, it is of great therapeutic interest because it is surgically accessible and can be provided with an auditory brainstem implant if cochlear implantation is no longer possible, e.g., due to a tumor resection of the auditory nerve. [17]. As a basis for these therapeutic approaches, knowledge about molecular details in neural stem cells (NSCs) and neurogenesis of the auditory system is necessary [2].

As previously described, NSC potential has been detected in CN into the adult stage [13]. The decrease in NSC potential over time indicates that hearing onset represents a critical phase for CN neurogenesis and NSC development [13]. The molecular processes affecting neurogenesis and NSC development in CN are mainly unknown. To investigate the molecular processes of NSC development and CN neurogenesis, mRNA abundance of neurogenic factors was compared between different time points depending on hearing capacity. For this purpose, Sprague Dawley rats were examined at p6 (not hearing), p12 (hearing onset), and p24 (hearing). After isolation of the RNA and reverse transcription into cDNA, mRNA abundance of 84 different genes essential for neurogenesis and NSC development was analyzed using a quantitative real-time polymerase chain reaction array (RT-qPCR). Heat maps, volcano plots, and analyses of individual genes were performed to identify mRNA abundance patterns of neurogenic factors and relevant and significant genes in maturing rat CN over time. The present study aimed to find an mRNA pattern during the critical postnatal phase of hearing onset within CN and identify neurogenic factors that may be crucial for regenerative neurogenesis.

## 2. Materials and Methods

### 2.1. Animal and Tissue Preparations

Before starting work, all surfaces were decontaminated with RNase Zap (Thermo-Fischer Scientific^®^, Grand Island, NE, USA), and all work utensils were sterilized. Postnatal Sprague Dawley rats (Charles-River^®^, Wilmington, MA, USA) at 6, 12, and 24 days of age were delivered on the appropriate days and immediately cervically dislocated and decapitated. Equal numbers of females and males were chosen for each age group. The cranial nerves were detached after the midsagittal opening of the skull and the removal of the bony portions. Subsequently, the brain, including the brainstem, was transferred to Neurobasal^®^ medium (Thermo-Fischer Scientific^®^, Grand Island, NE, USA) at room temperature. The cerebrum and cerebellum were carefully separated, and the meningeal tissue with blood vessels was detached from the brain stem with #5/45 preparation forceps (Dumont^®^, Montignez, Switzerland). Subsequently, CN was identified at the lateral brainstem under the microscope (OPMI1, Zeiss^®^, Oberkochen, Germany) and dissected bluntly with #5/45 preparation forceps (Dumont^®^, Montignez, Switzerland). Per animal, the two paired CN were transferred to DNA-, DNase-, RNase-, and pyrogen-free cryovials (Simport Scientific^®^, Saint-Mathieu-de-Beloeil, Canada) and placed immediately in liquid nitrogen for at least 15 min.

All experiments were conducted according to the national guidelines for the care and use of laboratory animals (§8) and carried out exclusively as organ removal. Removing organs from the animal after the sacrifice is subject to a notification requirement, as per § 6 Abs. 1 No. 4 (German Animal Welfare Act), but has not been and cannot be approved as an animal experiment.

The number of sacrificed animals per species per year has to be given to the local authorities. Accordingly, 12 sacrificed Sprague Dawley rats were reported to the “Regierung of Unterfranken” (Government Lower Franconia).

### 2.2. RNA Extraction from the Tissue of CN

The tissue was transferred from the cryovials into beads-filled tubes (Precellys^®^ Lysing Kit CK 14, Bertin, France). The CN of the animals of one age group were pooled in one bead tube (n = 4 animals and 8 CN per age group) without a medium. Pooling was necessary to generate enough mRNA for further analysis. Weighing of pooled CN was performed (Sartorius^®^ Handy M160, Goettingen, Germany). The pooled CN weighed less than 20 mg, regardless of age. Therefore, following the instructions of the RNeasy Mini Kit (Qiagen^®^, Venlo, The Netherlands), 350 µL of RLT buffer (Qiagen^®^, Venlo, The Netherlands) was added per tube. These were homogenized in two homogenizer steps (Precellys 24 DUAL^®^, Bertin, France) at 6000 rpm for 30 s each. A total of 350 µL of ethanol 70% (Thermo Fisher Scientific^®^, Waltham, MA, USA) was added to the resulting emulsion. Further steps were performed according to the instructions of the RNeasy Mini Kit (Qiagen^®^, Venlo, The Netherlands).

Subsequently, the extracted RNA was quantified using a spectrophotometer (NanoDrop One/One^c^, Thermo Fisher Scientific^®^, Waltham, MA, USA), and its purity (A260/A280) was determined. At postnatal day 6 (p6), animals had approximately 350 ng/mL RNA. p12 and p24 animals had about 750 ng/mL. Only RNA with an A260/A280 ratio of 2.0 ± 0.1 was used to synthesize complementary DNA (cDNA).

### 2.3. cDNA Synthesis from RNA by Reverse Transcription

Further steps were carried out according to the procedure described in the RT^2^ First Strand Kit (Qiagen^®^, Venlo, The Netherlands). For this purpose, a 10 µL DNA elimination mix was prepared with 500 ng RNA, Buffer GE, and RNase-free water per reaction. This was incubated for 5 min at 42 °C (Biometra Trio 30, Analytik Jena^®^, Jena, Germany) and then placed on ice for 1 min. Then, 10 µL reverse transcriptase mix with 2 µL RE3 reverse transcriptase (Qiagen^®^, Venlo, The Netherlands) was prepared and pipetted into the cooled DNA elimination mix. Incubation was repeated at 42 °C for 15 min and immediately followed by incubation at 95 °C for 5 min. Then, 91 µL RNase-free water was added per sample. Before the following steps, the samples were stored at −20 °C for a maximum of eight weeks.

### 2.4. Rat Neurogenesis RT^2^ Profiler^TM^ PCR Array

From the cDNA obtained in the previous step, 102 µL were mixed with 1.350 µL 2XRT2 SYBR Green Mastermix (Qiagen^®^, Venlo, The Netherlands) and 1.248 µL RNase free water. Then, 25 µL per well of this suspension was transferred to the 96-well Rat Neurogenesis RT2 Profiler^TM^ PCR Array (PARN-404ZC-12, Qiagen^®^, Venlo, The Netherlands), which was sealed. This array contains a primer set for 84 genes related to neurogenesis and NSCs. Furthermore, the array comprises 5 reference genes (*actin beta, beta-2-microglobulin, hypoxanthine phosphoribosyltransferase 1, lactate dehydrogenase A, ribosomal protein P1*), 3 reverse transcription controls (RTC), 3 PCR reproducibility controls (PPC), and 1 contamination control (GDC). Additional steps were performed on the real-time PCR system StepOnePlus^TM^ (Thermo Fisher Scientific^®^, Waltham, MA, USA), and the threshold values were identical for all analyses performed. The automated baseline option of the system was used as a baseline. PCR was performed at the following cycling conditions: 10 min at 95 °C for denaturation, 40 cycles at 95 °C for 15 s, and 60 °C for 1 min. The cycle threshold (Ct) determined this way was exported as a spreadsheet calculation from Microsoft^®^ Excel 2023 V16.70 (Microsoft Corporation, Redmond, WA, USA). For each age group, 3 replicates were performed from the pooled samples.

### 2.5. Data Analysis and Statistical Evaluation

All collected data were compiled using Microsoft^®^ Excel 2023 V16.70 (Microsoft Corporation, Redmond, WA, USA) spreadsheets. Raw Ct values were analyzed using the Qiagen^®^ GeneGlobe Data Analysis Web Portal (Qiagen, Venlo, The Netherlands). All samples passed the PCR array reproducibility test (ΔCt average RTC—average PPC ≤ 5), the reverse transcription efficiency test (Ct PPC of the three replicates within an array is 20 ± 2, and the average PPC CT values of any 2 arrays do not differ by more than 2) and the genomic DNA contamination test (Ct GDC ≥ 35). The gene *B2m* was included in the setup but was not used as a reference gene because the expression was unstable between ages. The expression of genes in the maturing CN was determined using 4 reference genes (*actin beta, hypoxanthine phosphoribosyltransferase 1, lactate dehydrogenase A*, and *ribosomal protein P*1). The selected reference genes showed stable expression in the rat model [18,19,20]. The geometric mean of multiple thoughtful selected and stable reference genes is recommended in the literature [21]. The expression of the chosen reference genes was stable between the different age groups, and their geometric mean differed less than one, as recommended by the Qiagen^®^ GeneGlobe Data Analysis Web Portal (Qiagen, Venlo, The Netherlands). Each gene was normalized with the geometric mean of the reference genes to obtain the ΔCt value. The ΔΔCt was calculated for each gene by subtracting the ΔCt value of the age group (n = 3) from the ΔCt value of the control age group (n = 3). The fold change for each gene from the age group to the control age group was calculated as 2^(−ΔΔCt)^. P-values were calculated using a student’s t-test based on the ΔCt values of the replicates for each gene in each age group compared to the control age group. The fold change data obtained this way was used to create a heat map containing all the genes represented on the RT-qPCR array plate, following instructions provided by the freely available web software Heatmapper [22]. Colors were selected according to the recommendations of current literature to provide the best possible access for color-blind readers [23]. The heat map was generated using complete linkage and Pearson correlation. The clustering methodology was chosen according to suggestions in the current literature [24]. The individual clusters without dendrogram and larger font of the gene names are attached in the supplementary material for better readability of the gene names. The volcano plot allows the evaluation of relevant changes in mRNA abundance in the context of their statistical significance. It represents on the *x*-axis the log base 2 of the fold change value of each gene and the *y*-axis the negative log base 10 of the *p*-value of the gene on the *y*-axis. The graphical representation of the volcano plots and the bar charts were created with GraphPad^®^ Prism 9.5.0 software (Graphpad Software Inc., San Diego, CA, USA). Data generated can be accessed under supplementary material. The final images were composed using Adobe^®^ InDesign CC 2023 v 18.1 software (Adobe Inc., San Jose, CA, USA). 

## 3. Results

### 3.1. mRNA Abundance Patterns of Neurogenic Factors Correlate with Age in Maturing CN

**Figure 1:**
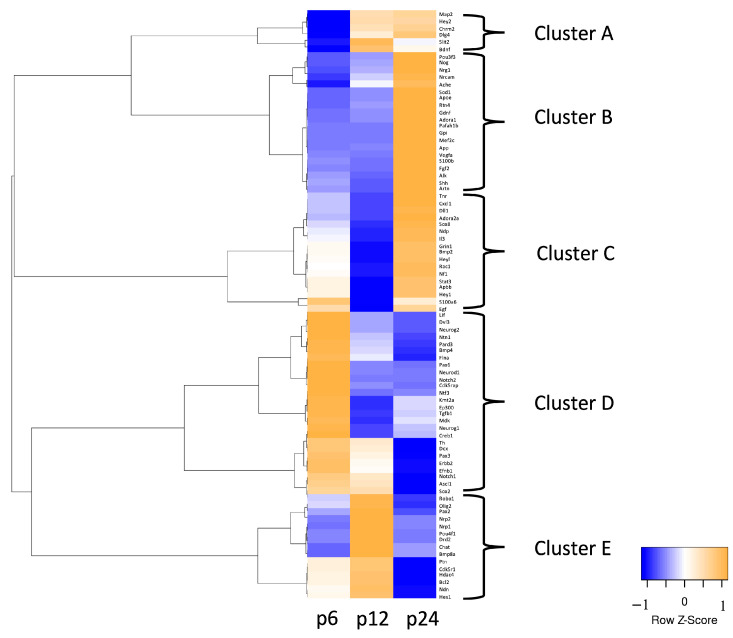
Before (p6), during (p12), and after (p24) the hearing onset, mRNA abundance of neurogenic factors in CN occurs in characteristic clusters. mRNA abundance profiles of neurogenic factors in CN are presented in a heat map with a hierarchical cluster-based dendrogram. Age groups are noted in the bottom margin, and gene names are in the heat map’s right margin. Complete linkage and Pearson correlation generated the dendrogram on the left margin. Mean fold changes from three independent experiments of all RT-qPCR analyzed mRNAs were used as input data. The Z-score visualizes the magnitude of gene expression with zero (white) equaling average expression over all samples and +/−1 indicating standard deviations from the mean. Blue fields indicate a lower mRNA abundance and orange fields indicate a higher one.

A heat map with a hierarchical-clustered dendrogram was generated to identify gene clusters in different age groups and therefore depending on hearing capacity (Figure 1). Clusters were defined based on the second branches of the dendrogram. Cluster A, which by previous definition would coincide with Cluster B, was defined subjectively for biological reasons. The genes in Cluster A already increase at hearing onset and not afterwards as in Cluster B. Therefore, Cluster A was defined as a separate cluster. This was made to emphasize the importance of the hearing onset and its influence on the mRNA level. The expression of neurogenic factors was shown to be age-dependent and thus to correlate well with hearing capacity. Hence, changes in a characteristic cluster of genes were shown at each time of investigation. Some mRNA clusters of particular interest will be described in detail in the following sections.

At p6, 7% of the genes examined (six genes) had a lower mRNA abundance at p6, followed by a higher mRNA abundance at p12 or p24. These genes were *microtubule-associated protein 2* (*Map2*), *hairy/enhancer-of-split related with YRPW motif 2* (*Hey2*), *cholinergic receptor muscarinic 2* (*Chrm2*), *discs large homolog 4* (*Dlg4*), *slit homolog 2* (*Slit2*). and *brain-derived neurotrophic factor* (*Bdnf*) (Figure 1, Cluster A).

In addition, 44% of the genes studied (37 genes) had the highest mRNA abundance at p24. Among those, two clusters of genes were identified, Cluster B and Cluster C. Cluster B showed low mRNA abundance at p6 and varying degrees of mRNA abundance at p12 before an increase at p24 (Figure 1, Cluster B). These genes were *POU class 3 homeobox 3* (*Pou3f3*), *noggin* (*Nog*), *neuregulin 1* (*Nrg1*), *neuronal cell adhesion molecule* (*Nrcam*), *acetylcholinesterase* (*Ache*), *superoxide dismutase 1* (*Sod1*), *apolipoprotein E* (*Apoe*), *reticulon 4* (*Rtn4*), *glial cell-derived neurotrophic factor* (*Gdnf*), *adenosine A1 receptor* (*Adora1*), *platelet-activating factor acetylhydrolase, isoform 1b* (*Pafah1b*), *glucose phosphate isomerase* (*Gpi*), *myocyte enhancer factor 2C* (*Mef2c*), *amyloid beta (A4) precursor protein* (*App*), *vascular endothelial growth factor* A (*Vegfa*), *S100 calcium-binding protein B* (*S100b*), *fibroblast growth factor 2* (*Fgf2*), *anaplastic lymphoma kinase* (*Alk*), *sonic hedgehog* (*Shh*), and *artemin* (*Artn*) (Figure 1, Cluster B). Cluster C showed intermediate mRNA abundance at p6, followed by a drastic drop at p12 before increasing at p24 (Figure 1, Cluster C). These genes were *tenascin R* (*Tnr*), *chemokine (C-X-C motif) ligand 1* (*Cxcl1*), *delta-like 1* (*Dll1*), *adenosine A2a receptor* (*Adora2a*), *SRY (sex determining region Y)-box* 8 (*Sox8*), *Norrie disease* (*Ndp*), *interleukin 3* (*Il3*), *glutamate receptor, ionotropic, N-methyl D-aspartate 1* (*Grin1*), *bone morphogenetic protein 2* (*Bmp2*), *hairy/enhancer-of-split related with YRPW motif-like* (*Heyl*), *ras-related C3 botulinum toxin substrate 1* (*Rac1*), *neurofibromin 1* (*Nf1*), *signal transducer and activator of transcription 3* (*Stat3*), *amyloid beta (A4) precursor protein-binding, family B, member 1 (Fe65)* (*Apbb1*), and *hairy/enhancer-of-split related with YRPW motif 1* (*Hey1*) (Figure 1, Cluster C). *S100 calcium-binding protein A6* (*S100a6*) and *epidermal growth factor* (*Egf*) were also included in Cluster C. These genes displayed a drastic decrease in mRNA abundance at p12, characteristic of this cluster. At p6 and p24, they showed a slight increase in mRNA abundance (Figure 1, Cluster C).

Analysis of Cluster D showed that 31% of the genes studied had a higher mRNA abundance before hearing onset at p6 than at p12 and p24 (Figure 1, Cluster D). These 26 genes showed a heterogeneous pattern at the following time points. Four subgroups were identified. For *leukemia inhibitory factor* (*Lif*), *disheveled dsh homolog 3* (*Dvl3*), *neurogenin 2* (*Neurog2*), *netrin 1* (*Ntn1*), *par-3 (partitioning defective 3) homolog* (*Pard3*), *bone morphogenetic protein 4* (*Bmp4*), and *filamin A* (*Flna*), there was a progression of the decrease in mRNA abundance with age (Figure 1, Cluster D). In contrast, *paired box 6* (*Pax6*), *neurogenic differentiation 1* (*Neurod1*), *notch homolog 2* (*Notch2*), *CDK5 regulatory subunit associated protein 2* (*Cdk5rap2*), and *neurotrophin 3* (*Ntf3*) had less mRNA abundance at p12 compared to p6 but did not change between p12 and p24 (Figure 1, Cluster D). In contrast, *myeloid/lymphoid or mixed-lineage leukemia 1* (*Kmt2a*), *E1A binding protein p300* (*Ep300*), *transforming growth factor, beta 1* (*Tgfb1*), *midkine* (*Mdk), neurogenin 1* (*Neurog1*), and *CAMP responsive element binding protein 1* (*Creb1*) underwent a drastic decrease in mRNA abundance between p6 and p12. Still, it displayed average levels at p24 (Figure 1, Cluster D). High mRNA abundance at p6 with intermediate mRNA abundance at p12 and a decrease in mRNA abundance at p24 was observed for *tyrosine hydroxylase* (*Th*), *doublecortin* (*DCX*), *paired box 3* (*Pax3*), *v-erb-b2 erythroblastic leukemia viral oncogene* (*Erbb2*), *ephrin B1* (*Efnb1*), *notch homolog 1* (*Notch1*), *achaete-scute complex homolog 1* (*Ascl1*), and *SRY (sex determining region Y)-box 2* (*Sox2*) (Figure 1, Cluster D).

Regarding Cluster E, mRNA abundance was the highest at p12 in 17% (15 genes) of the genes examined. Two subgroups were identified (Figure 1, Cluster E). Genes with selectively high mRNA abundance at p12 were *roundabout homolog 1* (*Robo1*), *oligodendrocyte lineage transcription factor 2* (*Olig2*), *paired box 2* (*Pax2*), *neuropilin 2* (*Nrp2*), *neuropilin 1* (*Nrp1*), *POU class 4 homeobox 1* (*Pou4f1*), *dopamine receptor D2* (*Drd2*), *choline acetyltransferase* (*Chat*), and *bone morphogenetic protein 8a* (*Bmp8a*) (Figure 1, Cluster E). The two subgroup displayed intermediate mRNA abundance before upregulation at p12 with a subsequent lower mRNA abundance at p24 (Figure 1, Cluster E). These genes were *pleiotrophin* (*Ptn*), *cyclin-dependent kinase 5, regulatory subunit 1* (*Cdk5r1*), *histone deacetylase 4* (*Hdac4*), *B-cell CLL/lymphoma 2* (*Bcl2*), *necdin* (*Ndn*), and *hairy and enhancer of split 1* (*Hes1*).

### 3.2. Identification of Neurogenic Factors with High Importance for NSC Development and Neurogenesis in Maturing CN

Having identified mRNA patterns in maturing CN, which vary in a hearing-correlating manner, volcano plots were generated to identify genes that exhibited both significant (*p* < 0.05) and relevant (fold change < 0.5 or >2) changes in mRNA abundance over time. Comparing all age groups, 38 genes were identified, whose mRNA abundance changes were significant and relevant.

**Figure 2:**
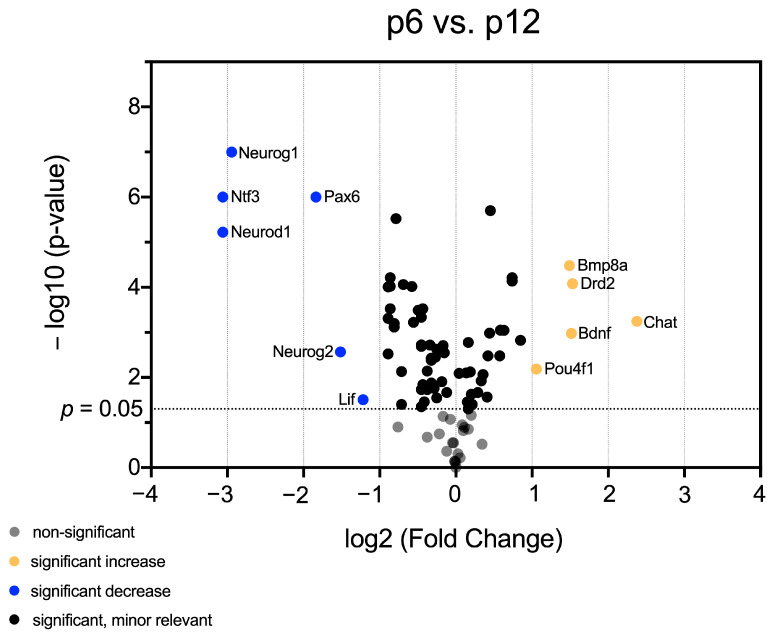
The volcano plot highlights significant and relevant differences in mRNA abundance of neurogenic factors in CN between p6 and p12. The *y*-axis displays the negative logarithm with base 10 of the *p*-value. The *p*-value is indicated with the dashed line at the corresponding position. Points below this dashed line have *p* > 0.05 and are shown in gray. The *x*-axis represents the logarithm with base 2 of fold change between p6 and p12. Points above the p-value are black if the fold change is less than two or more than 0.5 (log_2_ = 1/−1). Points that have a *p*-value < 0.0.5 and a fold change greater than two or less than 0.5 are shown in orange (higher mRNA abundance at p12 than at p6) and blue (lower mRNA abundance at p12 than at p6), respectively.

Comparison between p6 and p12 revealed a significant and relevant decrease in mRNA abundance of *Lif, Neurog2, Pax6, Neurod1, Ntf3*, and *Neurog1* at p12. In contrast, the mRNA abundance of *Chat, Pou4f1, Bdnf, Drd2*, and *Bmp8a* were significantly changed and relevantly higher at p12 than at p6 (Figure 2).

**Figure 3:**
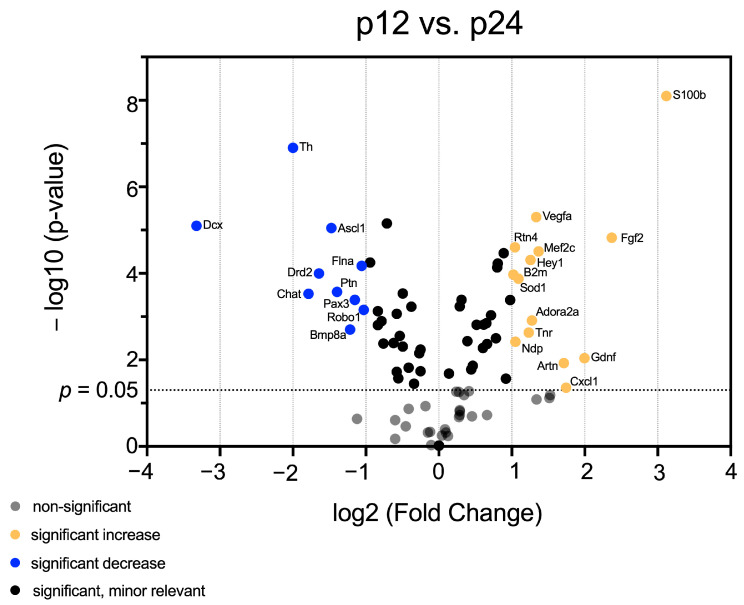
The volcano plot highlights significant and relevant differences in mRNA abundance of neurogenic factors in CN between p12 and p24. The *y*-axis displays the negative logarithm with base 10 of the *p*-value. The *p*-value is indicated with the dashed line at the corresponding position. Points below this dashed line have *p* > 0.05 and are shown in gray. The *x*-axis represents the logarithm with base 2 of fold change between p12 and p24. Points above the p-value are shown in black if the fold change is less than two or more than 0.5 (log_2_ = 1/−1). Points that have a *p*-value < 0.0.5 and a fold change greater than two or less than 0.5 are shown in orange (higher mRNA abundance at p24 than at p12) and blue (lower mRNA abundance at p24 than at p12), respectively.

The analysis of the age-stage comparison between p12 and p24 revealed age-dependent high and low mRNA abundance of neurogenic factors. Significantly changed and relevantly lower mRNA abundance at p24 than at p12 was detected for *Dcx, Th, Chat, Drd2, Ascl1, Flna, Ptn, Paax3, Robo1,* and *Bmp8a*. In contrast, *Rtn4, Vegfa, Mef2c, Hey1, B2m, Sod1, Adora2a, Tnr, Ndp, Artn, Cxcl1, Gdnf, Fgf2*, and *S100b* had significantly changed and relevantly higher mRNA abundance in p24 than p12 (Figure 3).

**Figure 4:**
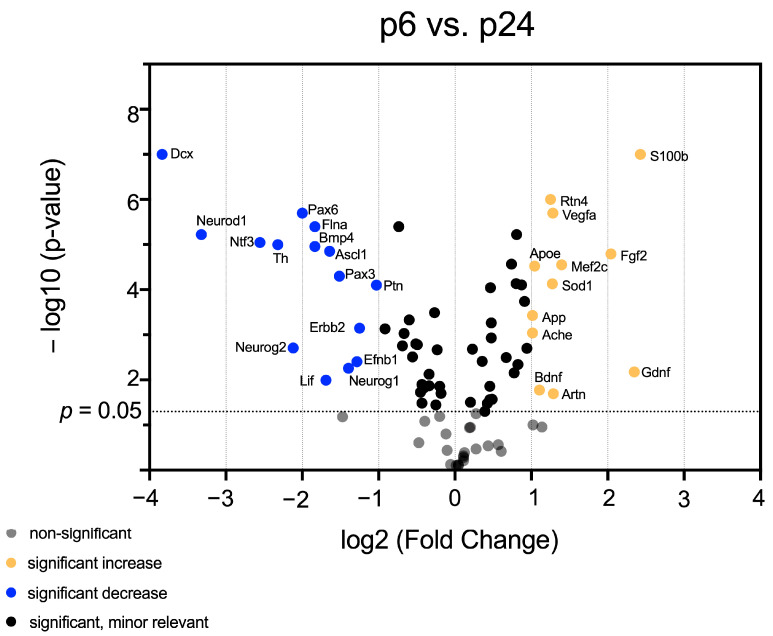
Volcano plot highlights significant and relevant differences in mRNA abundance of neurogenic factors in CN between p6 and p24. The *y*-axis displays the negative logarithm with base 10 of the *p*-value. The *p*-value is indicated with the dashed line at the corresponding position. Points below this dashed line have *p* > 0.05 and are shown in gray. The *x*-axis represents the logarithm with base 2 of fold change between p6 and p24. Points above the p-value are shown in black if the fold change is less than two or more than 0.5 (log_2_ = 1/−1). Points that have a *p*-value < 0.0.5 and a fold change greater than two or less than 0.5 are shown in orange (higher mRNA abundance at p24 than at p6) and blue (lower mRNA abundance at p24 than at p6), respectively.

The comparison of age groups p6 and p24 showed significant and relevant changes in mRNA abundance of most of the same factors previously identified in the comparison of age groups p6 and p12. The factors *Dcx, Neurod1, Ntf3, Th, Pax6, Flna, Bmp4, Ascl1, Ptn, Erbb2, Neurog2, Lif, Efnb1*, and *Neurog1* displayed a significantly changed and relevantly lower mRNA abundance at p24 than at p6. The genes *Artn, Bdnf, Gdnf, Ache, App, Sod1, Apoe, Mef2c, Fgf2, Vegfa, Rtn4*, and *S100b* had a significantly changed and relevantly higher mRNA abundance at p24 than at p6 (Figure 4).

### 3.3. In-Depth Analysis of Selected Critical Neurogenic Factors in Maturing CN

To analyze and highlight the importance of individual neurogenic factors, which show significant and relevant differences in mRNA abundance, their 2^-−ΔCt^ values were examined over time. For this purpose, genes were selected whose relative expression changes were significant within the relevantly altered cohort (fold change < 0.5 or >2) and showed the most pronounced changes. Genes with a fold change of >4 or <0.25 (log2 fold change = 2/−2) were examined at one of the analyzed stages. Four genes met this criterion with higher mRNA abundance over time. Namely, *Gdnf, Fgf2, Chat*, and *S100b* were analyzed. Additionally, for the genes with lower mRNA abundance over time, *Dcx, Neurog1, Neurog2, Neurod1, Th*, and *Ntf3* met the fold change <4 criterion.

**Figure 5:**
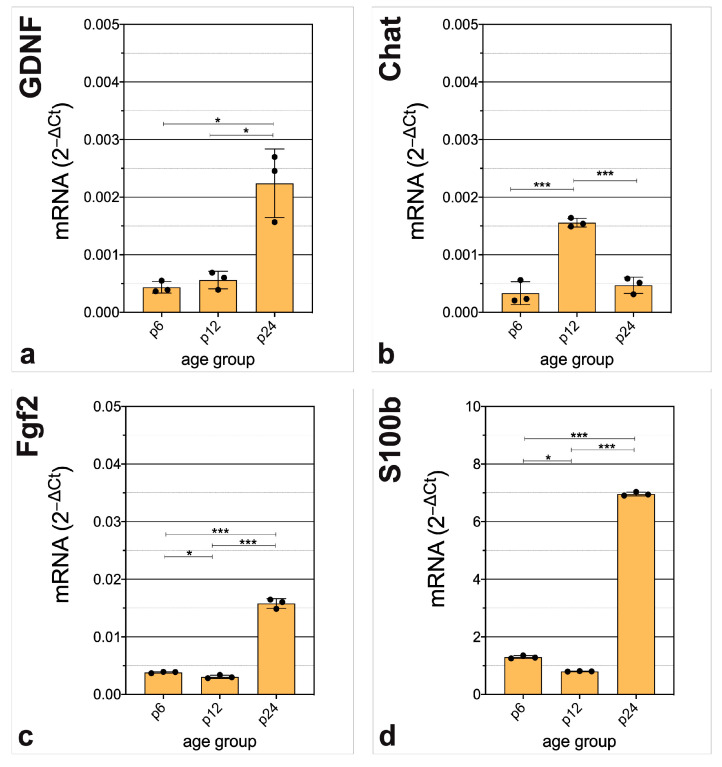
Highlighting genes critical for CN neurogenesis with a significant and relevant increase in mRNA abundance. Genes were chosen, whose relative expression changes were significant (*p <* 0.05) and had a fold change > 4 between two age groups. The critical neurogenic factors (**a**) *Gdnf,* (**c**) *Fgf2* and (**d**) *S100b* display significantly higher amounts of mRNA at p24 than at p6 and p12, whereas (**b**) *Chat* has the highest mRNA abundance at p12. The bar charts represent the mean with standard deviation (SD); each point represents one of the three independently performed experiments, n = 3; asterisks indicate the significance level, * *p* < 0.05, *** *p <* 0.001.

Essential genes in CN neurogenesis displayed a significant increase in mRNA abundance over time. mRNA abundance of *Gdnf* was significantly higher at p24 than at p6. There was no relevant difference between p6 and p12 (p6 vs. p12, *p* = 0.3004; p12 vs. p24, *p* = 0.0091; p6 vs. p24, *p* = 0.0066) (Figure 5a). Interestingly, *Chat* showed significantly higher mRNA abundance at p12 than at p6 with significantly lower mRNA abundance at p24 than at p12 (p6 vs. p12, *p* = *p* < 0.001; p12 vs. p24, *p* < 0.001; p6 vs. p24, *p* = 0.3818) (Figure 5b). After a significantly but slightly lower mRNA abundance at p12 than at p6, there was a pronounced significantly higher mRNA abundance of *Fgf2* at p24 than at p12 (p6 vs. p12, *p* = 0.0132; p12 vs. p24, *p* < 0.001; p6 vs. p24, *p* < 0.001) (Figure 5c). Similarly, the mRNA abundance of *S100b* was shown to be significantly altered. After a slightly higher mRNA abundance at p6 than at p12, there was a significantly higher mRNA abundance at p24 (p6 vs. p12, *p* = 0.0087; p12 vs. p24, *p* < 0.001; p6 vs. p24, *p* < 0.001) (Figure 5d).

**Figure 6:**
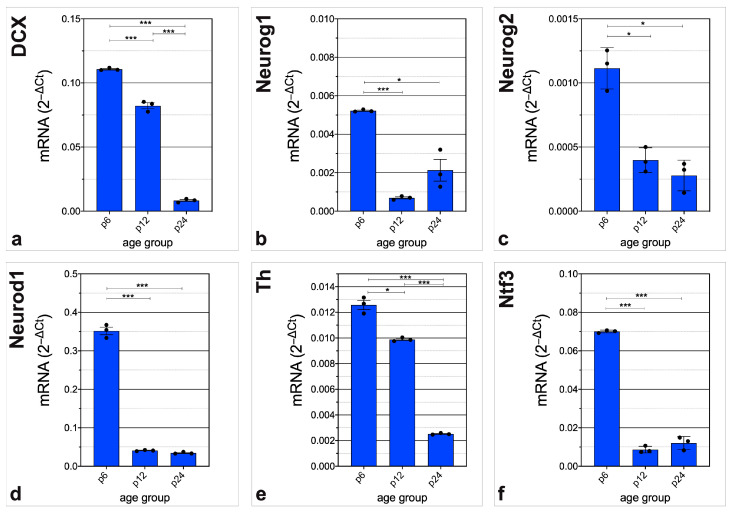
Highlighting genes critical for CN neurogenesis with a significant and relevant decrease in mRNA abundance. Genes were chosen, whose relative expression changes were significant (*p <* 0.05) and had a fold change < 0.25 between two age groups. The critical neurogenic factors (**a**) *Dcx,* (**b**) *Neurog1,* (**c**) *Neurog2,* (**d**) *Neurod1*, (**e**) *Th*, and (**f**) *Ntf3* have the highest mRNA abundance at the hearing onset (p6) with significant decrease at p12 and p24. The bar charts represent the mean with standard deviation (SD); each point represents one of the three independently performed experiments, n = 3; asterisks indicate the significance level, * *p <* 0.05, *** *p <* 0.001.

Essential genes in CN neurogenesis displayed significant decrease in mRNA abundance over time. *Dcx* displayed a significant decrease in mRNA abundance over time. There was a significantly lower mRNA abundance at p12 and p24 than at p6 (p6 vs. p12, *p <* 0.001; p12 vs. p24, *p = p <* 0.001; p6 vs. p24, *p = p <* 0.001) (Figure 6a). The mRNA abundance of *Neurog1* and *Neurog2* were significantly lower at p12 and p24 than at p6 (*Neurog1*, p6 vs. p12, *p = p <* 0.001; p12 vs. p24, *p =* 0.0648; p6 vs. p24, *p =* 0.0054) (*Neurog2*, p6 vs. p12, *p =* 0.0057; p12 vs. p24, *p =* 0.2471; p6 vs. p24, *p =* 0.0059) (Figure 6b,c). *Neurod1* had similar differences in mRNA abundance. mRNA abundance was significantly lower at p12 and p24 that at p6 (p6 vs. p12, *p <* 0.001; p12 vs. p24, *p =* 0.0583; p6 vs. p24, *p <* 0.001) (Figure 6d). *Th* mRNA abundance was significantly lower at p12 and p24 than at p6 (p6 vs. p12, *p =* 0.0069; p12 vs. p24, *p <* 0.001; p6 vs. p24, *p <* 0.001) (Figure 6e). *Ntf3* mRNA abundance was significantly lower at p12 and p24 than at p6 (p6 vs. p12, *p <* 0.001; p12 vs. p24, *p =* 0.2011; p6 vs. p24, *p <* 0.001) (Figure 6f).

## 4. Discussion

**Figure 7:**
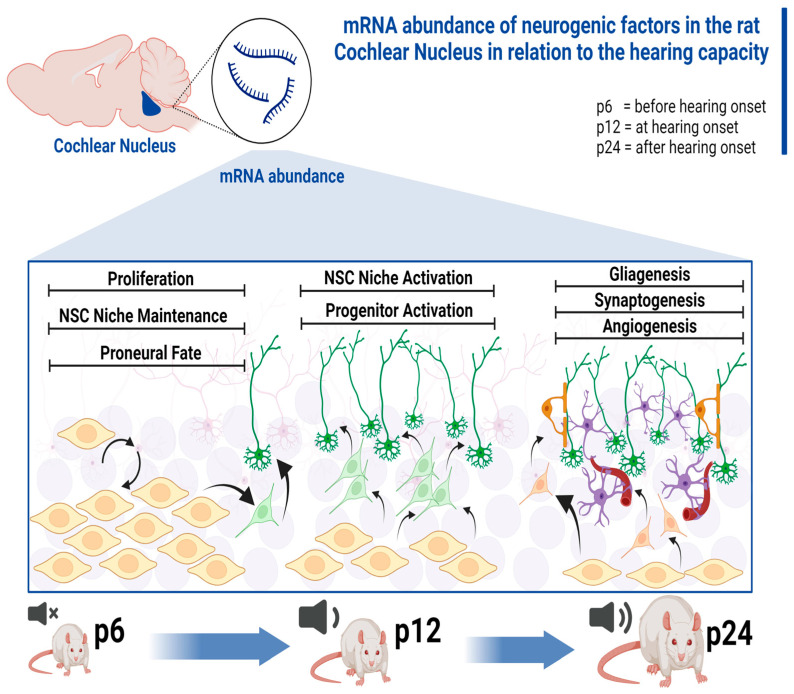
Graphical representation of CN neurogenesis at the mRNA level in relation to auditory capacity. At each age group, there is a characteristic mRNA abundance of neurogenetic factors. Prior to hearing onset (p6), genes that influence proliferation, proneural fate, and neural stem cell niche maintenance experience an increase in mRNA level. Around hearing onset (p12), activation of the neural stem cell niche and progenitor cells occurs at the mRNA level. After hearing onset (p24), increased mRNA abundance was detected in genes related to gliagenesis, synaptogenesis, and angiogenesis. ). Figure was created with BioRender.com (accessed on 30^th^ August 2023).

mRNA levels of neurogenic factors were analyzed in rat CN before hearing onset (p6), at hearing onset (p12), and after hearing onset (p24). This analysis revealed characteristic mRNA abundance patterns over time about hearing (Figure 7). Furthermore, it was shown that a differentially pronounced significance of the differences in mRNA abundance of the individual neurogenic factors was present. Specific gene(s) groups shape the neurogenesis of rat CN at crucial time points of the auditory system on the mRNA level. Analysis of these factors demonstrated that the changes in their mRNA level were closely correlated to the results obtained from in vitro studies [13]. A detailed analysis of the mRNA level of neurogenic factors at the crucial stages of the maturing CN has been demonstrated, to the best of our knowledge, for the first time.

### 4.1. bHLH Family of Transcription Factors and Pax Gene Family Play Essential Roles in CN Neurogenesis and Regulation of NSC Pool on mRNA Level

Analysis of significantly and relevantly altered mRNA abundance of neurogenic factors identified that the basic helix-loop-helix (*bHLH*) family of transcription factors and the *Pax* gene family assume a critical role in CN NSC niche regulation at the mRNA level.

Genes of the *bHLH* transcription factor family and the *Pax* gene family are strongly represented in Cluster D, which has as a standard feature a high mRNA abundance before hearing onset (Figure 1, Cluster D). *Pax3* and *Pax6* are important in maintaining NSCs and their differentiation into neurons [25]. *Pax3* regulates migration and differentiation in precursor cell populations [26]. Removal of *Pax6* reduces NSC self-renewal and results in early neurogenesis [27]. mRNA abundance of *Pax3* and *Pax6* was significant and relevantly higher at p6 than at p24 (Figure 4). *Pax2* plays an essential role in developing the inner ear. A high correlation between *Pax2* expression and the proliferation of chicken hair cells has been reported in the literature [28]. The selective elevation of *Pax2* mRNA at hearing onset and its influence on the inner ear highlight its importance for the maturation of sensory proneural cells in the auditory system. These results indicate an essential role of *Pax* genes in maintaining CN NSC niche before hearing onset.

The *bHLH* genes *Neurog1, Neurog2*, *Ascl1,* and *Neurod1* play a central role in the development and maturation of the auditory pathway [29]. These genes displayed significant and relevant changes at mRNA levels (Figure 4 and Figure 6b–d). They contribute to the proliferation, cell cycle, and differentiation of neurons [30,31]. They are essential for neuron survival and crucial in forming internuclear connections of the auditory pathway [29,31,32,33]. *Neurog1, Neurog2,* and *Ascl1* function to induce proneural differentiation while suppressing gliogenesis [34]. These results emphasize the proneural influence of these *bHLH* transcription factors on the NSC pool before hearing onset.

*HeyL, Hey1, Hes1*, and *Hey2* are members of the *Hey/Hes* family, which are also *bHLH* transcription factors. *Hey1* and *Hey2* promote neural precursor cell maintenance in the brain and negatively regulate neuronal *bHLH* genes [35]. mRNA abundance of both genes was elevated at p24, with *Hey2* already upregulated around hearing onset (Figure 1, Cluster A and C). *Hey1* mRNA abundance was significant and relevantly higher at p24 than at p12 (Figure 3). *HeyL* mRNA increased at p24 (Figure 1, Cluster C). This is interesting because *HeyL*, on the other hand, promotes neuronal differentiation from neural progenitor cells by inhibiting other *Hey/Hes* genes [36]. These expression patterns suggest that these genes play a critical role in maintaining the CN NSC niche after activation at hearing onset.

Mutual interactions were found between the previously described factors about neurogenesis and the NSC niche. Interestingly, *Pax3* was shown to regulate both *bHLH* genes *Hes1* and *Neurog2* [37]. In mouse embryos with nonfunctional *Pax3* mutant, transcripts of *Hes1* and *Neurog2* and, consequently, neurogenesis and NSC development were reduced. Therefore, two crucial functions are attributed to *Pax3* in this context—first, the maintenance of stem cell character secured by the *bHLH* gene *Hes1* [37]. *Hes1* ensures proper maintenance of the stem cell niche, and when missing, premature neurogenesis occurs [38]. On the other hand, the *bHLH* gene *Neurog2* initiates the development of the neuronal lineage of NSCs. *Neurog2* assumes a critical role in sensory neurogenesis [39].

The results of this study suggest that at the mRNA level, the CN NSC niche is maintained by *Pax* genes before hearing onset. At the same time, the results indicate that stimulation by activating bHLH genes (Neurog1, Neurog2, Neurod1, and Ascl1) stimulates proneural progenitor cells before hearing onset. As described in 4.2, there is an increase in neurogenic factors at the mRNA level at hearing onset that activates the CN NSC niche (Figure 1, Cluster E). At the same time, there is a decrease in proneural mRNA and activating *bHLH* genes at the hearing onset (Figure 2). *bHLH* genes that suppress and thus maintain the CN NSC niche (*Hey/Hes*) mostly experience an increase in mRNA after hearing onset (Figure 1, Clusters A, C, and E). Therefore, transcription factors of the *Pax* family and the *bHLH* family may represent potential targets for future gene therapies to manipulate CN Neurogenesis and NSC niche.

### 4.2. NSC Niche-Activating Transcripts Are Elevated at the Hearing Onset

At hearing onset (p12), factors that promote a proneural fate of NSCs and the survival of newly formed neurons and progenitor cells have a higher mRNA abundance (Figure 7). The genes whose mRNA abundance started to increase at p12 are labeled Cluster A, and genes with mRNA abundance higher at p12 are labeled Cluster E (Figure 1, Cluster A, and Cluster E).

Cluster A contained *Map2, Hey2, Chrm2, Dlg4, Slit2,* and *Bdnf* (Figure 1, Cluster A). Most of these genes had the highest mRNA abundance at p24 and are therefore discussed at 4.4. *Bdnf* has a vital role in the survival of newly formed cells, and its mRNA abundance was significant and relatively higher at p12 than at p6 (Figure 2) [40]. Interestingly, *Bdnf* mRNA was expressed in the early postnatal days and is downregulated with maturation in the rat inner ear [41]. These results indicate that on the mRNA level, *Bdnf* has an essential role in developing and surviving neurons in the auditory system.

Cluster E consisted of genes relevant for stimulation of NSC niche (*Chat, Drd2,* and *Bmp8a*), regulation of newly formed progenitor and neurons (*Pax2, Nrp1, Nrp2, Cdk5r1,* and *Hes1*), regulation of gliogenesis (*Ndn* and *Olig2*), neuroprotection and survival of new neurons (*Hdac4, Ptn,* and *Bcl2*), and axogenesis (*Robo1, Pou4f1*) [42,43,44,45,46,47,48,49,50,51,52,53,54].

mRNA abundance of *Bmp8a, Drd2, Pou4f1*, and *Chat* were significantly and relevantly higher at p12 than at p6 (Figure 2). *Bmps* are activated in NSCs that enter a neuronal fate [55]. *Bmp8a* provides proneural differentiation in hippocampal NSCs, which is critical, and Pou4f1 plays a crucial role in neuronal differentiation and survival [49]. *Chat* and *Drd2* provide stimulation of NSC niche and proneural progenitor cells [56,57]. The interplay of these factors indicates that activation of CN NSC niche and neuronal differentiation of NSCs occurs at hearing onset. The acoustic stimulus that starts at the hearing onset is a potential trigger for these changes [58]. The changes in *Chat* mRNA abundance were significant and pronounced higher at p12 than at p6 (Figure 5b). Therefore, this neurogenic factor was highlighted. It was shown by Chat+ immunoreaction detection in the chicken that efferent neurons pulling from CN to the cochlea are 70% cholinergic [59]. The increase in *Chat* mRNA at p12 possibly represents the increased formation of cochlear efferents in response to auditory input triggered by hearing onset.

Around the hearing onset, genes stimulating NSCs to a proneural fate with expansion and protection of these cells had high mRNA abundance. This indicates that neuronal input from the cochlea is accompanied by the stimulation of genes in CN, which promote the formation and stabilization of signal-transducing neuronal structures. Interestingly, factors with crucial influence on axogenesis were also upregulated. Axonal growth and axon-path-finding, together with proneural regulation, shaped the expression pattern of neurogenetic factors at the time of hearing onset in CN. *Slit1* and *Robo1* are essential in axogenesis in embryonal CN [60].

Interestingly, auditory stimulus promotes the differentiation and maturation of neurons from NSCs in CN. Here, a connection with the clusterin pathway has been described [58]. The results of this study, which examined the physiological development of CN maturation in response to hearing, made comparable findings at the mRNA level at the time of hearing onset.

The results of this study suggest that proneural stimulation occurs at the mRNA level at hearing onset at the expense of the NSC pool. The increased mRNA of neurogenic factors stimulating the NSC niche suggests that the critical balance between proliferation and differentiation is shifting towards differentiation at hearing onset. In vitro, a decrease in neurospheres was observed up to p12 with stable neurosphere formation at a low level [13]. Neurospheres are a correlate for mitotic self-renewal and proliferation of NSCs [61]. Analysis of the cell division and proliferative marker BrdU showed high cell division activity up to p12 with a decrease and stable low levels from p20 [13]. These results suggest a close correlation between the mRNA level and the results expressed at the protein level. The characteristic changes at the mRNA level of neurogenic factors in CN reflect the changes in the in vitro results. This is interesting because other brain sections with NSC niches have similarly been found to have decreasing NSC capacity in vitro. A prominent example is the dentate gyrus of the hippocampus. Here, a decrease in new neurons forming was observed [62,63]. Using a similar methodology, no significant change in neurogenic factors mRNA abundance in the dentate gyrus was detected. mRNA abundance of the neurogenic factors was stable over time [64].

### 4.3. mRNA of Neurogenic Factors Stimulating Proliferation, Neuronal Migration, and Proneural Differentiation Is Abundant before Hearing Onset

Before hearing onset (p6), mRNA abundance of genes that promote proliferation, neuronal migration, and proneural differentiation was higher than at p12 and p24 (Figure 1, Cluster D) (Figure 7). Four subgroups were identified, depending on the mRNA levels at subsequent ages.

The first subgroup had a slight decrease in mRNA abundance at hearing onset with a significant reduction at p24 and contained the *genes Lif, Dvl3, Neurog2, Ntn1, Pard3, Bmp4,* and *Flna* (Figure 1, Cluster D). *Ntn1, Pard3,* and *Flna* promote the maintenance of self-renewing progenitor cells [65,66,67]. *Neurog2* had a significant and relevantly higher mRNA abundance at p6 compared to subsequent ages and promotes proneural differentiation (Figure 2 and Figure 4) [30]. NSCs transplanted into the inner ears of guinea pigs with degenerated spiral ganglions differentiated into neurons after transduction with *Neurog2* [68]. These results indicate that *Neurog2* is critical for developing newly formed neurons in spiral ganglions. *Bmp4* and *Lif* were expressed similarly and promoted gliogenesis [69,70]. *Dvl3* is essential for cochlea and brain maturation at early stages [71]. Another subgroup, whose mRNA levels remained consistently low beginning at p12, included genes that promote maintenance of the neural stem cell pool (*Pax6, Notch2,* and *Cdk5rap2*) and a proneural fate of progenitor cells (*Ntf3* and *Neurod1*) (Figure 1, Cluster D) [25,33,72,73,74]. The changes in mRNA abundance of *Ntf3* in CN over time were significant and relevant (Figure 6f). The essential function of *Ntf3* for forming neurons in the spiral ganglion was demonstrated [75]. These results suggest that *Ntf3* plays a vital role at the mRNA level in forming new neurons in the auditory system. *Kmt2a, Ep300, Tgfb1, Mdk, Neurog1,* and *Creb1* formed another subgroup (Figure 1, Cluster D). After a decrease in mRNA level at hearing onset (p12), this subgroup had intermediate mRNA abundance at p24. This subgroup consisted of genes that promote a proneural fate of progenitor cells and maintain a progenitor cell pool [31,76,77,78,79]. *Mdk* promotes NSC migration [80]. Another subgroup consisted of genes promoting neuronal migration (*Dcx*), maintaining of the NSC pool (*Pax3, Efnb1, Notch1,* and *Sox2*), and differentiation (*Th, Erbb2* and *Ascl1*) (Figure 1, Cluster D) [25,73,81,82,83,84,85,86]. *Th* had significant and slightly lower mRNA abundance at p12 than at p6. Subsequently, there was a substantial drop in mRNA abundance at p24 (Figure 6e). In the spiral ganglion, protein expression studies of Tyrosin-hydroxylase revealed a peak before hearing onset with a decline at the hearing onset. It was suggested that *Th* indicates the hearing onset in the spiral ganglion [87]. mRNA abundance of *Dcx,* which promotes neuronal migration, was significant and relevantly higher at p6 than at p24 and significant and relevantly higher at p12 than at p24 (Figure 3 and Figure 4). Thus, the crucial decrease at the mRNA level of *Dcx* occurs after hearing onset (Figure 6a). The protein DCX has been shown to play a critical role in maturing neurons in other neurogenic areas. Its expression decreased with age [88]. Additionally, the dorsal part of CN expresses DCX [89]. These findings indicate that *Dcx* is essential for neuronal migration in developing CN at the mRNA level before and at hearing onset.

Analysis of genes with higher mRNA abundance before hearing onset (p6) in CN indicates that this development point is critical for mRNA-level neurogenesis. On the one hand, proliferative factors and genes that are relevant for the maintenance of a self-renewing progenitor or stem cell pool, and on the other hand, factors that are essential for the migration and formation of new neurons shape CN neurogenesis before hearing onset. This suggests a critical balance between proliferation, differentiation, and regulation of the NSC pool before hearing onset.

Additionally, the genes with the most pronounced changes in mRNA abundance showed high biological relevance to the auditory pathway, as the critical influence of these genes and their gene products was found in other nuclei of the auditory pathway. This indicates that NSC development and neurogenesis along the auditory pathway share certain mRNA-level intranuclear similarities.

### 4.4. Regulators of Gliogenesis, Neuritogenesis, Synaptogenesis, and Angiogenesis Are Prominent at the Transcriptional Level of CN after Hearing Onset

Clusters B and C contain genes whose mRNA levels increased after hearing onset. The genes in Cluster B had a low mRNA abundance before hearing onset. In contrast, Cluster C is characterized by mRNA of these genes being intermediate before hearing onset and decreased at hearing onset (Figure 1, Cluster B and C). Genes whose mRNA levels increase after hearing onset largely influence gliogenesis, neuritogenesis, and angiogenesis.

The *genes S100b, Sod1, Chrm2, Sox8, Bmp4, Erbb2, Nf1, Gpi,* and *Bmp2* are relevant for gliogenesis and development of astrocytes [70,86,90,91,92,93,94,95]. mRNA abundance of these genes is high after hearing onset (p24) (Figure 1, Cluster B and C). *S100b* mRNA abundance was significantly and relevantly higher (Figure 5d). *S100b* mRNA is largely localized in astroglial cells. Interestingly, S100-positive neurons were detected at a very high density in the auditory system, especially in CN [96]. In rodents, there is an increase in *S100b* expression within the first three postnatal weeks, indicating that astrocytes significantly regulate glial proliferation and synaptic plasticity [97]. These results suggest that the increase in *S100b* mRNA abundance after hearing onset has a crucial influence on the gliogenesis and synaptogenesis of CN.

Similarly, the mRNA level of genes influencing neuritogenesis and synaptogenesis was increased. *Mef2c, Rtn4, Grin1*, *Pafah1b1, Il3, Map2, Nrcam, Apoe, Ache, App, Dlg4, Gdnf*, *Alk,* and *Artn* play essential roles in neuritogenesis, and synapse formation and mRNA levels of these genes are higher at p24 (Figure 1, Cluster B and C) [98,99,100,101,102,103,104,105,106,107,108,109,110,111]. The timing of maturation and development of synaptogenesis is consistent with studies of other brain regions. In rat motor-sensory cortex, the density of synapses increases rapidly from about 14 days onward and shows a peak between 20 and 30 days [112]. *Gdnf* had a significantly and relevantly higher mRNA abundance at p24 than at p6 and p12 (Figure 5a). *Gdnf* has been detected up to the adult stage in the spiral ganglion and other sections of the cochlea and is suggested to promote neuritogenesis and protect neuronal cells [101,113]. An age-dependent comparison has yet to be made in these studies. Interestingly, a similar mRNA pattern of *Gdnf* has been demonstrated in the rat cerebellum [114]. These results suggest the potential importance of *Gdnf* for the protection and survival of auditory neurons.

A subset of genes (*Vegfa, Ndp*, *Adora1,* and *Adora2a*) whose mRNA abundance is increased after hearing onset (p24) has an essential influence on angiogenesis and blood–brain barrier formation (Figure 1, Cluster B and C) [115,116,117]. The increase in mRNA level of these genes was significant and relevant between p12 and p24 (Figure 4). Interestingly, cerebral capillary diameters increase at p24 in healthy rats [118]. These results suggest that *Adora2a, Vegfa*, and *Ndp* influence angiogenesis in maturing CN.

Interestingly, mRNA abundance of genes influencing neural progenitor cells and NSC niche is increased after hearing onset. Some of these genes (*Nrg1, Stat3, Rac1, Pou3f3, Nog, Shh,* and *Dll1*) displayed no significant and relevant change in mRNA abundance (Figure 1, Cluster B and C) [73,119,120,121,122,123,124,125]. The mRNA levels of several of these genes (*B2m, Tnr, Cxcl1, Hey1, Fgf2*) were significantly and relevantly altered over time (Figure 3). *B2m* is a pro-aging factor and inhibits neurogenesis [126]. *Tnr* regulates NSC niche and neurogenesis [127]. *Hey1* promotes NSC maintenance [35]. *Cxcl1* inhibits the proliferation of NSC niche [128]. *Fgf2* mRNA abundance was significantly and relevantly higher at p24 than at p6 and p12 (Figure 5c). *Fgf2* has an essential function in neurons of the peripheral and central auditory pathways [129]. *Egf* and *Fgf2* ensure the survival and proliferation of NSCs into the adult stage [130]. The proliferation of neural progenitor cells occurs after cerebral ischemia by administration of *Egf* and *Fgf2* [131]. *Fgf2* has a crucial influence on the NSC niche by suppressing astrocytic differentiation and preserving dormancy [132]. In the context of gene therapy for treating Alzheimer’s disease, the therapeutic potential of Fgf2 by stimulating NSCs in neurodegenerative disorders has already been demonstrated [133]. These results indicate that after hearing onset (p24) NSC niche is regulated at the mRNA level by different factors promoting the maintenance of the NSC niche.

### 4.5. Limitations of the Study

Exclusively the level of mRNA of neurogenic factors was investigated. An analysis of proteomics is necessary to verify whether changes at the mRNA level correlate with expression at the protein level in vivo in the rat. This study aimed to determine whether and to what extent changes in selected neurogenic factors are apparent at the mRNA level. The chosen genes were classified according to their biological importance and the project’s research question. Transcripts subsequently showed a highly relevant biological significance in the auditory system (4.1 and 4.3). In addition, 2^(−ΔCt)^ values rather than fold change were analyzed to assess RNA content. Thus, evaluating the change’s expression of the mRNA abundance level is possible. All relevant altered genes in 3.2 were also analyzed and evaluated for neurogenesis. Identifying NSC niche or single cell groups showing appropriate neurogenic potential is impossible with this methodology. What kind of cells express these neurogenic factors and thus provide the required milieu for NSCs remains unknown. In this respect, single-cell RNA sequencing would be a relevant extension of the current work. This study will hopefully serve as a basis for additional investigations of the neurogenic capacity of CN at the transcriptional and other levels.

## 5. Conclusions

In summary, neurogenic mRNA patterns correlating with hearing capacity, as well as factors with essential impact on CN neurogenesis, were identified. Before hearing onset (p6), mRNA abundance of factors promoting NSC niche maintenance, proneural differentiation, and proliferation were elevated. At hearing onset (p12), mRNA levels of genes that promote activation of the NSC niche increased. After hearing onset (p24), genes affecting synaptogenesis, angiogenesis, and gliogenesis had high mRNA abundance. Significantly and relevantly altered genes were identified. Changes at the mRNA level reflected in vitro changes in CN NSC capacity. Furthermore, the relevant altered neurogenic factors closely correlated to the maturation and neurogenesis of other nuclear areas of the auditory pathway. The results of this study may contribute to a better understanding of molecular processes of auditory pathway neurogenesis.

## Data Availability

The data used to support the findings of this study are included within the article.

## Supplementary Materials

The following supporting information can be downloaded at: https://www.mdpi.com/article/10.3390/life13091858/s1. Table S1: Gene Table, Table S2: Average Ct, Table S3: Average Delta(Ct), Table S4: 2^(−Avg.(Delta(Ct)), Table S5: Fold Change, Table S6: *p*-value, Figure S1: The individual clusters without dendrogram and larger font of the gene names.
